# Quarterly Report of the Obstetric Department of the Philadelphia Dispensary, for 1st, 2d, and 3d Months, 1844

**Published:** 1844-06-01

**Authors:** Joseph Warrington

**Affiliations:** 229 Vine street


*Quarterly Report of the Obstetric Department of the Philadelphia Dispensary, for* 1*st*, 2*d*, *and* 3*d months*, 1844. By Joseph Warrington, M. D.

Forty-two women have been delivered: forty at or near full term; one at the 7th or 8th month; and one at the 6th month of utero-gestation.

The average duration of labour in thirty-five was fifteen hours; the extremes being two and seventy-two hours.

The average amount of time required for the spontaneous delivery of the placenta, in thirty-six cases, was 18 minutes; the extremes being 5 and 60 minutes.

There were two instances in which the uterus remained in a state of engorgement, to a size reaching nearly to the umbilicus, for several days after delivery. They gradually diminished under the use of moderate frictions, with mild embrocations, bandages, and the usual purgation. These engorgements excited no constitutional disturbance.

Manual assistance was required for the delivery of the placenta in four cases, in consequence of the manner in which it presented the centre of its disc to the os uteri.

Ergot was given in one case to re-excite contractions of the uterus, which had become quiescent after its orifice had dilated sufficiently to admit of the passage of the child very readily. It was followed by rapid delivery of a large and healthy male child.

Hemorrhage occurred during the third stage of labour in two cases. It was instantly arrested upon the delivery of the placenta, which was effected by the introduction of one hand into the uterus, and the firm application of the other upon the abdomen.

There were two cases of metro-peritonitis. One occurring in a patient, who was easily delivered of a child which had died in utero some time before it reached the eighth month of development. In the other case, an early labour was followed by profuse hemorrhage before the delivery of the placenta. They both recovered under the use of antiphlogistic treatment.

Forty-three children, 30 males and 13 females, have been born; one woman having twins.

The cephalic extremity of the fetal ellipse presented in forty-one instances; the pelvis once; and both feet once.

Of the forty-one cephalic presentations, nineteen were noted to be in the first position; six in the second; one in the fourth; and four in the fifth. There was also one case in which the chin presented to the left acetabulum. The breech and the feet, each presented in the second position.

In one case of original fifth position of the head, spontaneous rotation occurred, and the occiput emerged under the arch of the pubis.

In another case of fifth position, the descent of the head appeared to be facilitated by pressing the forehead upwards under the arch, that the occiput could more readily slide down upon the plane of the sacrum, coccyx, and perinæum.

Forceps was used in two cases: in one, to assist the descent of the head through the cavity of the pelvis, in a feeble woman, who had formerly been treated with ergot; in one, to extract the head in a footling case, in which the whole labour had continued seventy-two hours.

One of the twin children had convulsions come on a few days after birth; it recovered under the use of small doses of calomel and carbonate of soda.

One child had hemorrhage from the umbilicus after the spontaneous separation of the cord. By the surgical skill of Dr. Goddard, the umbilical vessels were secured, but the child sunk from capillary hemorrhage; the blood, in a very fluid, uncoagulable state, oozing from the eyes, nose, mouth, and ears.

Five children were found to have died before delivery. In three of these, the cuticle was peeled off at several points; in one other, the cord prolapsed without pulsation, and the breech instantly followed; in the fifth case, there was no sign of life at the end of labour; the presentation being footling, and the head extracted by forceps; the first stage of the labour had occupied more than two days.

In the premature delivery at six months, the child, though alive at the moment of extrusion, soon afterwards expired.

The neck was found encircled by the cord in four cases; twice in two instances, three times in one, and five times in another; which so impeded the delivery, that it was not completed till, with the aid of the hand, the breech and the feet were brought down. The child was asphixied, but by careful attention it was resuscitated.

Sixteen of the patients who have been under our care, have received the aid of the Nurse Society, and for five others we obtained the assistance of the funds of the Philadelphia Lying-in Charity; and, by a recent arrangement with regard to these two institutions, it is hoped that more extended usefulness of these important collateral aids may be effected.

229 *Vine street, Fourth mo.* 1844.

---

Strumous Conjunctivitis.—Dr. Hocken recommends the application of the nitrate of silver to the *outside* of the lids, and De La Garde and Lanyon, who have adopted the method, approve highly of it.

